# valr: Reproducible genome interval analysis in R

**DOI:** 10.12688/f1000research.11997.1

**Published:** 2017-06-29

**Authors:** Kent A. Riemondy, Ryan M. Sheridan, Austin Gillen, Yinni Yu, Christopher G. Bennett, Jay R. Hesselberth

**Affiliations:** 1 RNA Bioscience Initiative, University of Colorado School of Medicine, Aurora, CO, 80045, USA; 2 Department of Biochemistry and Molecular Genetics, University of Colorado School of Medicine, Aurora, CO, 80045, USA; 3 ComAnalyzeIT LLC, Fort Collins, CO, 80525, USA

**Keywords:** Genomics, Intervals, BEDtools, reproducibility, R, RStudio

## Abstract

New tools for reproducible exploratory data analysis of large datasets are important to address the rising size and complexity of genomic data. We developed the valr R package to enable flexible and efficient genomic interval analysis. valr leverages new tools available in the ”tidyverse”, including dplyr. Benchmarks of valr show it performs similar to BEDtools and can be used for interactive analyses and incorporated into existing analysis pipelines.

## Introduction

A routine bioinformatic task is the analysis of the relationships between sets of genomic intervals, including the identification of DNA variants within protein coding regions, annotation of regions enriched for nucleic acid binding proteins, and computation of read density within a set of exons. Command-line tools for interval analysis such as BEDtools ^1^ and BEDOPS ^2^ enable analyses of genome-wide datasets and are key components of analysis pipelines. Analyses with these tools commonly combine processing intervals on the command-line with visualization and statistical analysis in R. However, the need to master both the command-line and R hinders exploratory data analysis, and the development of reproducible research workflows built in the RMarkdown framework.

Existing R packages developed for interval analysis include IRanges ^3^, bedr ^4^, and GenometriCorr ^5^. IRanges is a Bioconductor package that provides interval classes and methods to perform interval arithmetic, and is used by many Bioconductor packages. bedr is a CRAN-distributed package that provides wrapper R functions to call the BEDtools, BEDOPS, and tabix command-line utilities, providing out-of-memory support for interval analysis. Finally, GenometriCorr provides a set of statistical tests to determine the relationships between interval sets using IRanges data structures. These packages provide functionality for processing and statistical inference of interval data, however they require a detailed understanding of S4 classes ( IRanges) or the installation of external command-line dependencies ( bedr). Additionally, these packages do not easily integrate with the recent advances provided by the popular tidyverse suite of data processing and visualization tools (e.g. dplyr, purrr, broom and ggplot2) ^6^. We therefore sought to develop a flexible R package for genomic interval arithmetic built to incorporate new R programming, visualization, and interactivity features.

## Methods

### Implementation

valr is an R package that makes extensive use of dplyr, a flexible and high-performance framework for data manipulation in R ^7^. Additionally, compute intensive functions in valr are written in C++ using Rcpp to enable fluid interactive analysis of large datasets ^8^. Interval intersections and related operations use an interval tree algorithm to efficiently search for overlapping intervals ^9^. BED files are imported and handled in R as data_frame objects, requiring minimal pre or post-processing to integrate with additional R packages or command-line tools.

### Operation

valr is distributed as part of the CRAN R package repository and is compatible with Mac OS X, Windows, and major Linux operating systems. Package dependencies and system requirements are documented in the valr CRAN repository.

## Use cases

To demonstrate the functionality and utility of valr, we present a basic tutorial for using valr and additional common use cases for genomic interval analysis.

### Basic usage

***Input data.*** valr provides a set of functions to read BED, BEDgraph, and VCF formats into R as convenient tibble (tbl) data_frame objects. All tbls have chrom, start, and end columns, and tbls from multi-column formats have additional pre-determined column names. Standards methods for importing data (e.g. read.table, readr::read_tsv) are also supported provided the constructed dataframes contain the requisite column names ( chrom, start, end). Additionally, valr supports connections to remote databases to access the UCSC and Ensembl databases via the db_ucsc and db_ensembl functions.



                        library(valr)

                        # function to retrieve path to example data

                        bed_filepath <- valr_example(
                        "3fields.bed.gz"
                        )
read_bed(bed_filepath)

                        #> # A tibble: 10 x 3
#>    chrom  start    end
#>    <chr>  <int>  <int>
#>  1  chr1  11873  14409
#>  2  chr1  14361  19759
#>  3  chr1  14406  29370
#>  4  chr1  34610  36081
#>  5  chr1  69090  70008
#>  6  chr1 134772 140566
#>  7  chr1 321083 321115
#>  8  chr1 321145 321207
#>  9  chr1 322036 326938
#> 10  chr1 327545 328439
                    




                        #using URL

                        read_bed(
                        "https://github.com/rnabioco/valr/raw/master/inst/extdata/3fields.bed.gz"
                        )

                        #> # A tibble: 10 x 3
#>    chrom  start    end
#>    <chr>  <int>  <int>
#>  1  chr1  11873  14409
#>  2  chr1  14361  19759
#>  3  chr1  14406  29370
#>  4  chr1  34610  36081
#>  5  chr1  69090  70008
#>  6  chr1 134772 140566
#>  7  chr1 321083 321115
#>  8  chr1 321145 321207
#>  9  chr1 322036 326938
#> 10  chr1 327545 328439
                    


***Example of combining valr tools.*** The functions in valr have similar names to their BEDtools counterparts, and so will be familiar to users of the BEDtools suite. Also, similar to pybedtools ^10^, a python wrapper for BEDtools, valr has a terse syntax. For example, shown below is a demonstration of how to find all intergenic SNPs within 1 kilobase of genes using valr. The BED files used in the following examples are described in the Data Availability section.



                        library(dplyr)


                        snps <- read_bed(valr_example(
                        "hg19.snps147.chr22.bed.gz"
                        ), 
                        n_fields = 
                        6
                        )
genes <- read_bed(valr_example(
                        "genes.hg19.chr22.bed.gz"
                        ), 
                        n_fields = 
                        6
                        )


                        # find snps in intergenic regions

                        intergenic <- bed_subtract(snps, genes)

                        # distance from intergenic snps to nearest gene

                        nearby <- bed_closest(intergenic, genes)


                        nearby %>%
  select(starts_with(
                        "name"
                        ), .overlap, .dist) %>%
  filter(abs(.dist) < 
                        1000
                        )

                        #> # A tibble: 285 x 4
#>         name.x            name.y .overlap .dist
#>          <chr>             <chr>    <int> <int>
#>  1   rs2261631             P704P        0  -267
#>  2 rs570770556             POTEH        0  -912
#>  3 rs538163832             POTEH        0  -952
#>  4   rs9606135            TPTEP1        0  -421
#>  5  rs11912392 ANKRD62P1-PARP4P3        0   104
#>  6   rs8136454          BC038197        0   355
#>  7   rs5992556              XKR3        0  -455
#>  8 rs114101676              GAB4        0   473
#>  9  rs62236167             CECR7        0   261
#> 10   rs5747023             CECR1        0  -386
#> # ... with 275 more rows
                    


**Figure 1.**
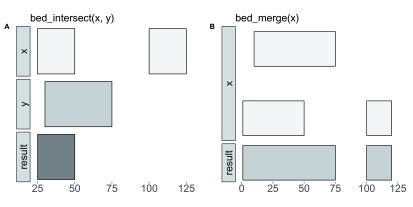
Visualizing interval operations in valr with bed_glyph().

***Visual documentation.*** By conducting interval arithmetic entirely in R, valr is also an effective teaching tool for introducing interval analysis to early-stage analysts without requiring familiarity with both command-line tools and R. To aid in demonstrating the interval operations available in valr, we developed the bed_glyph() tool which produces plots demonstrating the input and output of operations in valr in a manner similar to those found in the BEDtools documentation. Shown below is the code required to produce glyphs displaying the results of intersecting x and y intervals with bed_intersect(), and the result of merging x intervals with bed_merge() ( Figure 1).



                        x <- tibble::tribble(
  ~chrom, ~start, ~end,
  
                        "chr1"
                        , 
                        25
                        ,      
                        50
                        ,
  
                        "chr1"
                        , 
                        100
                        ,     
                        125

                        )

y <- tibble::tribble(
  ~chrom, ~start, ~end,
  
                        "chr1"
                        , 
                        30
                        ,      
                        75

                        )

bed_glyph(bed_intersect(x, y))
                    


And this glyph illustrates bed_merge():



                        x <- tibble::tribble(
  ~chrom, ~start, ~end,
  
                        "chr1"
                        ,       
                        1
                        ,      
                        50
                        ,
  
                        "chr1"
                        ,       
                        10
                        ,     
                        75
                        ,
  
                        "chr1"
                        ,       
                        100
                        ,    
                        120

                        )

bed_glyph(bed_merge(x))



***Grouping data.*** The group_by function in dplyr can be used to execute functions on subsets of single and multiple data_frames. Functions in valr leverage grouping to enable a variety of comparisons. For example, intervals can be grouped by strand to perform comparisons among intervals on the same strand.



                        x <- tibble::tribble(
  
                        ~chrom, ~start, ~end, ~strand,
  
                        "chr1"
                        , 
                        1
                        ,       
                        100
                        ,  
                        "+"
                        ,
  
                        "chr1"
                        , 
                        50
                        ,      
                        150
                        ,  
                        "+"
                        ,
  
                        "chr2"
                        , 
                        100
                        ,     
                        200
                        ,  
                        "-"

                        )


                        y <- tibble::tribble(
  
                        ~chrom, ~start, ~end, ~strand,
  
                        "chr1"
                        , 
                        50
                        ,      
                        125
                        ,  
                        "+"
                        ,
  
                        "chr1"
                        , 
                        50
                        ,      
                        150
                        ,  
                        "-"
                        ,
  
                        "chr2"
                        , 
                        50
                        ,      
                        150
                        ,  
                        "+"

                        )


                        # intersect tbls by strand

                        x <- group_by(x, strand)
y <- group_by(y, strand)

bed_intersect(x, y)

                        #> # A tibble: 2 x 8
#>   chrom start.x end.x strand.x start.y end.y strand.y .overlap
#>   <chr>   <dbl> <dbl>    <chr>   <dbl> <dbl>    <chr>    <int>
#> 1  chr1       1   100        +      50   125        +       50
#> 2  chr1      50   150        +      50   125        +       75



Comparisons between intervals on opposite strands are done using the flip_strands() function:



                        x <- group_by(x, strand)

y <- flip_strands(y)
y <- group_by(y, strand)

bed_intersect(x, y)

                        #> # A tibble: 3 x 8
#>   chrom start.x end.x strand.x start.y end.y strand.y .overlap
#>   <chr>   <dbl> <dbl>    <chr>   <dbl> <dbl>    <chr>    <int>
#> 1  chr2     100   200        -      50   150        -       50
#> 2  chr1       1   100        +      50   150        +       50
#> 3  chr1      50   150        +      50   150        +      100



Both single set (e.g. bed_merge()) and multi set operations will respect groupings in the input intervals.

***Column specification.*** Columns in BEDtools are referred to by position:



                        # calculate the mean of column 6 for intervals in ‘b‘ that overlap with ‘a‘

                        bedtools map -a a.bed -b b.bed -c 6 -o mean



In valr, columns are referred to by name and can be used in multiple name/value expressions for summaries.



                        # calculate the mean and variance for a ‘value‘ column

                        bed_map(a, b, 
                        .mean = 
                        mean(value), 
                        .var = 
                        var(value))


                        # report concatenated and max values for merged intervals

                        bed_merge(a, 
                        .concat = 
                        concat(value), 
                        .max = 
                        max(value))



***API.*** The major functions available in valr are shown in Table 1.

**Table 1.** An overview of major functions available in valr.

Function Name	Purpose
**Reading Data**	
read_bed	Read BED files
read_bedgraph	Read bedGraph files
read_narrowpeak	Read narrowPeak files
read_broadpeak	Read broadPeak files
**Interval Transformation**	
bed_slop	Expand interval coordinates
bed_shift	Shift interval coordinates
bed_flank	Create flanking intervals
bed_merge	Merge overlapping intervals
bed_cluster	Identify (but not merge) overlapping intervals
bed_complement	Create intervals not covered by a query
**Interval Comparison**	
bed_intersect	Report intersecting intervals from x and y tbls
bed_map	Apply functions to selected columns for overlapping intervals
bed_subtract	Remove intervals based on overlaps
bed_window	Find overlapping intervals within a window
bed_closest	Find the closest intervals independent of overlaps
**Randomizing intervals**	
bed_random	Generate random intervals from an input genome
bed_shuffle	Shuffle the coordinates of input intervals
**Interval statistics**	
bed_fisher, bed_ projection	Calculate significance of overlaps between two sets of intervals
bed_reldist	Quantify relative distances between sets of intervals
bed_absdist	Quantify absolute distances between sets of intervals
bed_jaccard	Quantify extent of overlap between two sets of intervals
**Utilities**	
bed_glyph	Visualize the actions of valr functions
bound_intervals	Constrain intervals to a genome reference
bed_makewindows	Subdivide intervals
bed12_to_exons	Convert BED12 to BED6 format
interval_spacing	Calculate spacing between intervals
db_ucsc, db_ensembl	Access remote databases

### Summarizing interval coverage across genomic features

This demonstration illustrates how to use valr tools to perform a “meta-analysis” of signals relative to genomic features. Here we analyze the distribution of histone marks surrounding transcription start sites, using H3K4Me3 Chip-Seq data from the ENCODE project.

First we load packages and relevant data.



                        bedfile <- valr_example(
                        "genes.hg19.chr22.bed.gz"
                        )

                        genomefile <- valr_example(
                        "hg19.chrom.sizes.gz"
                        )

                        bgfile  <- valr_example(
                        "hela.h3k4.chip.bg.gz"
                        )


                        genes <- read_bed(bedfile, 
                        n_fields =
                         6
                        )

                        genome <- read_genome(genomefile)

                        y <- read_bedgraph(bgfile)



Then, we generate 1 bp intervals to represent transcription start sites (TSSs). We focus on + strand genes, but - genes are easily accommodated by filtering them and using bed_makewindows() with reversed window numbers.



                        # generate 1 bp TSS intervals, "+" strand only

                        tss <- genes %>%
  filter(strand == 
                        "+"
                        ) %>%
  mutate(
                        end = 
                        start + 
                        1
                        )


                        # 1000 bp up and downstream

                        region_size <- 
                        1000

                        # 50 bp windows

                        win_size <- 
                        50


                        # add slop to the TSS, break into windows and add a group

                        x <- tss %>%
  bed_slop(genome, 
                        both = 
                        region_size) %>%
  bed_makewindows(genome, win_size)

x

                        #> # A tibble: 13,530 x 7
#>    chrom    start      end      name score strand .win_id
#>    <chr>    <int>    <int>     <chr> <chr>  <chr>   <int>
#>  1 chr22 16161065 16161115 LINC00516     3      +       1
#>  2 chr22 16161115 16161165 LINC00516     3      +       2
#>  3 chr22 16161165 16161215 LINC00516     3      +       3
#>  4 chr22 16161215 16161265 LINC00516     3      +       4
#>  5 chr22 16161265 16161315 LINC00516     3      +       5
#>  6 chr22 16161315 16161365 LINC00516     3      +       6 
#>  7 chr22 16161365 16161415 LINC00516     3      +       7
#>  8 chr22 16161415 16161465 LINC00516     3      +       8
#>  9 chr22 16161465 16161515 LINC00516     3      +       9
#> 10 chr22 16161515 16161565 LINC00516     3      +      10
#> # ... with 13,520 more rows



Now we use the .win_id group with bed_map() to calculate a sum by mapping y signals onto the intervals in x. These data are regrouped by .win_id and a summary with mean and sd values is calculated.



                        # map signals to TSS regions and calculate summary statistics.

                        res <- bed_map(x, y, 
                        win_sum = 
                        sum(value, 
                        na.rm = 
                        TRUE)) %>%
  group_by(.win_id) %>%
  summarize(
                        win_mean = 
                        mean(win_sum, 
                        na.rm = 
                        TRUE),
             
                         win_sd = 
                        sd(win_sum, 
                        na.rm = 
                        TRUE))
             

                        res

                        #> # A tibble: 41 x 3
#>    .win_id win_mean    win_sd
#>      <int>    <dbl>     <dbl>
#>  1       1 100.8974  85.83423
#>  2       2 110.6829  81.13521
#>  3       3 122.9070  99.09635
#>  4       4 116.2800  96.30098
#>  5       5 116.3500 102.33773
#>  6       6 124.9048  95.08887
#>  7       7 122.9437  94.39792
#>  8       8 127.5946  91.47407
#>  9       9 130.2051  95.71309
#> 10      10 130.1220  88.82809
#> # ... with 31 more rows
                    


**Figure 2.**
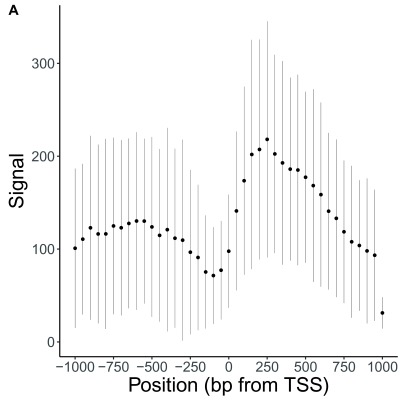
Meta-analysis of signals relative to genomic features with valr. ( **A**) Summarized coverage of human H3K4Me3 Chip-Seq coverage across positive strand transcription start sites on chromosome 22. Data presented +/- SD.

Finally, these summary statistics are used to construct a plot that illustrates histone density surrounding TSSs ( Figure 2).



                        library(ggplot2)


                        x_labels <- seq(-region_size, region_size, 
                        by = 
                        win_size ∗ 
                        5
                        )

                        x_breaks <- seq(
                        1
                        , 
                        41
                        , 
                        by = 
                        5
                        )


                        sd_limits <- aes(
                        ymax = 
                        win_mean + win_sd, 
                        ymin = 
                        win_mean - win_sd)


                        p <- ggplot(res, aes(
                        x = 
                        .win_id, 
                        y = 
                        win_mean)) +
  
                        geom_point(
                        size = 
                        0.25
                        ) + geom_pointrange(sd_limits, 
                        size = 
                        0.1
                        ) +
  
                        scale_x_continuous(
                        labels = 
                        x_labels, 
                        breaks = 
                        x_breaks) +
  
                        xlab(
                        "Position (bp from TSS)"
                        ) + ylab(
                        "Signal"
                        ) +
  theme_classic()
                    


### Interval statistics

Estimates of significance for interval overlaps can be obtained by combining bed_shuffle(), bed_random() and the sample_ functions from dplyr with interval statistics in valr.

Here, we examine the extent of overlap of repeat classes (repeatmasker track obtained from the UCSC genome browser) with exons in the human genome (hg19 build, on chr22 only, for simplicity) using the jaccard similarity index. bed_jaccard() implements the jaccard test to examine the similarity between two sets of genomic intervals. Using bed_shuffle() and replicate() we generate a data_frame containing 100 sets of randomly selected intervals then calculate the jaccard index for each set against the repeat intervals to generate a null-distribution of jaccard scores. Finally, an empirical p-value is calculated from the null-distribution.



                        library(tidyverse)


                        repeats <- read_bed(valr_example(
                        "hg19.rmsk.chr22.bed.gz"
                        ), 
                        n_fields = 
                        6
                        )
genome <- read_genome(valr_example(
                        "hg19.chrom.sizes.gz"
                        ))
genes <- read_bed12(valr_example(
                        "hg19.refGene.chr22.bed.gz"
                        ))

                        # convert bed12 to bed with exons

                        exons <- bed12_to_exons(genes)


                        # function to repeat interval shuffling

                        shuffle_intervals <- function(n, .data, genome) {
  
                        replicate(n, bed_shuffle(.data, genome), 
                        simplify = 
                        FALSE) %>%
    
                        bind_rows(
                        .id = 
                        "rep"
                        ) %>%
    
                        group_by(rep) %>% nest()

                        }


                        nreps <- 
                        100
                    




                        shuffled <- shuffle_intervals(
                        n = 
                        nreps, repeats, genome) %>%
  
                        mutate(
                        jaccard = 
                        data %>%
            
                        map(bed_jaccard, repeats) %>%
            
                        map_dbl(
                        "jaccard"
                        ))

                        shuffled

                        #> # A tibble: 100 x 3
#>      rep                  data      jaccard
#>    <chr>                <list>        <dbl>
#>  1     1 <tibble [10,000 x 6]> 0.0003388967
#>  2     2 <tibble [10,000 x 6]> 0.0004965988
#>  3     3 <tibble [10,000 x 6]> 0.0002974843
#>  4     4 <tibble [10,000 x 6]> 0.0006899870
#>  5     5 <tibble [10,000 x 6]> 0.0004678412
#>  6     6 <tibble [10,000 x 6]> 0.0001726937
#>  7     7 <tibble [10,000 x 6]> 0.0004694941
#>  8     8 <tibble [10,000 x 6]> 0.0004660410
#>  9     9 <tibble [10,000 x 6]> 0.0006846643
#> 10    10 <tibble [10,000 x 6]> 0.0002143829
#> # ... with 90 more rows


                        obs <- bed_jaccard(repeats, exons)

                        obs

                        #> # A tibble: 1 x 4
#>    len_i   len_u    jaccard     n
#>    <dbl>   <dbl>      <dbl> <dbl>
#> 1 112123 4132109 0.02789139   805


                        pvalue <- sum(shuffled$jaccard >= obs$jaccard) + 
                        1 
                        /(nreps + 
                        1
                        )

                        pvalue

                        #> [1] 0.00990099
                    


### Benchmarking against bedtools

**Figure 3.**
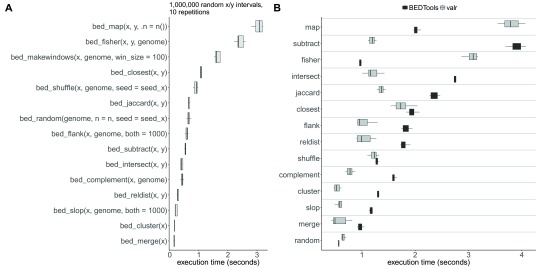
Performance of valr functions. ( **A**) Timings were calculated by performing 10 repetitions of indicated functions on data frames preloaded in R containing 1 million random 1 kilobase x/y intervals generated using bed_random(). ( **B**) Timings for executing functions in BEDtools v2.25.0 or equivalent functions in valr using the same interval sets as in ( **A**) written to files. All BEDtools function outputs were written to /dev/null/, and were timed using GNU time. Timings for valr functions in ( **B**) include times for reading files using read_bed() functions and were timed using the microbenchmark package.

In order to ensure that valr performs fast enough to enable interactive analysis, key functionality is implemented in C++. To test the speed of major valr functions we generated two data_frames containing 1 million randomly selected 1 kilobase intervals derived from the human genome (hg19). Most of the major valr functions complete execution in less than 1 second, demonstrating that valr can process large interval datasets efficiently ( Figure 3A).

We also benchmarked major valr functions against corresponding commands in BEDtools. valr operates on data_frames already loaded into RAM, whereas BEDtools performs file-reading, processing, and writing. To compare valr against BEDtools we generated two BED files containing 1 million randomly selected 1 kilobase intervals derived from the human genome (hg19). For valr functions, we timed reading the table into R (e.g. with read_bed()) and performing the respective function. For BEDtools commands we timed executing the command with the output written to / dev/null. valr functions performed similarly or faster than BEDtools commands, with the exception of bed_map and bed_fisher ( Figure 3B).

### Reproducible reports and interactive visualizations

Command-line tools like BEDtools and bedops can be incorporated into reproducible workflows (e.g., with snakemake ^11^), but it is cumbersome to transition from command-line tools to exploratory analysis and plotting software. RMarkdown documents are plain text files, amenable to version control, which provide an interface to generate feature rich PDF and HTML reports that combine text, executable code, and figures in a single document. valr can be used in RMarkdown documents to provide rapid documentation of exploratory data analyses and generate reproducible work-flows for data processing. Moreover, new features in RStudio, such as notebook viewing, and multiple language support enable similar functionality to another popular notebook platform jupyter notebooks.

Additionally, valr seamlessly integrates into R shiny ^12^ applications allowing for complex interactive visualizations relating to genomic interval analyses. We have developed a shiny application (available on Gitub) that explores ChiP-Seq signal density surrounding transcription start sites and demonstrates the ease of implementing valr to power dynamic visualizations.

## Summary

valr provides a flexible framework for interval arithmetic in R/Rstudio. valr functions are written with a simple and terse syntax that promotes flexible interactive analysis. Additionally by providing an easy-to-use interface for interval arithmetic in R, valr is also a useful teaching tool to introduce the analyses necessary to investigate correlations between genomic intervals, without requiring familiarity with the command-line. We envision that valr will help researchers quickly and reproducibly analyze genome interval datasets.

## Data and software availability

The valr package includes external datasets stored in the inst/extdata/ directory that were used in this manuscript. These datasets were obtained from the ENCODE Project ^13^ or the UCSC genome browser ^14^. BED files were generated by converting the UCSC tables into BED format. BED and BEDgraph data was only kept from chromosome 22, and was subsampled to produce file sizes suitable for submission to the CRAN repository. The original raw data is available from the following sources:

**hela.h3k4.chip.bg.gz** SRA record: SRR227441, ENCODE identifier: ENCSR000AOF

**hg19.refGene.chr22.bed.gz** ftp://hgdownload.soe.ucsc.edu/goldenPath/hg19/database/refGene.txt.gz

**hg19.rmsk.chr22.bed.gz** ftp://hgdownload.soe.ucsc.edu/goldenPath/hg19/database/rmsk.txt.gz

**hg19.chrom.sizes.gz** ftp://hgdownload.soe.ucsc.edu/goldenPath/hg19/database/chromInfo.txt.gz

**genes.hg19.chr22.bed.gz** ftp://hgdownload.soe.ucsc.edu/goldenPath/hg19/database/refGene.txt.gz

**hg19.snps147.chr22.bed.gz** ftp://hgdownload.soe.ucsc.edu/goldenPath/hg19/database/snp147.txt.gz

valr can be installed via CRAN using install.packages("valr").

valr is maintained at http://github.com/rnabioco/valr.

Latest valr source code is available at http://github.com/rnabioco/valr.

The latest stable version of source code is at: https://github.com/rnabioco/valr/archive/v0.3.0.tar.gz

Archived source code at the time of publication: http://doi.org/10.5281/zenodo.815403 ^15^

License: MIT license.
