# Supplementary material for: valr: Reproducible genome interval analysis in R
Source: F1000Res. 2017 Jun 29;6:1025. [Version 1] doi: 10.12688/f1000research.11997.1 (PMC5506536; doi:10.12688/f1000research.11997.1)
Supplement: Peer review history

# Peer review: Referee response for version 1

**DOI:** 10.5256/f1000research.12975.r24101

**Published:** 2017-07-10

**Authors:** Ryan K. Dale

**Affiliations:** 1 Laboratory of Cellular and Developmental Biology, National Institute of Diabetes and Digestive and Kidney Diseases, National Institutes of Health, Bethesda, MD, USA

The authors describe a package for manipulating genomic interval data in R using principles from the "tidyverse" for the data structures and API. This sets it apart from existing tools such as GenomicRanges or bedr which have their own ways of storing and manipulating data. As a result, valr should be easier to pick up and integrate with the rest of the R ecosystem, and the “tidyverse” in particular. Illustrative examples give the reader a taste for the package while highlighting the novel features.

 In general, this looks to be a very useful tool. The code quality is excellent and it is great to see so many tests including the addition of regression tests as issues are identified.

 My comments are very minor:

  

- Group-by code listing: comment "# intersect tbls by strand" should be "# group tbls by strand"
- Bioconductor might be a more appropriate repository than CRAN
- Description of in-memory usage: I see from the software documentation that BAM and VCF will be supported in the future, and the documentation explicitly mentions that valr operates on data in-memory. The section comparing with BEDTools briefly mentions the in-memory aspect, but it would be helpful to be clearer about memory usage in the manuscript, especially as users attempting to use large BAM files may run out of memory. 
- This is just a suggestion for improvement: Over the years, numerous bugs from corner cases have been found and handled in BEDTools. It would greatly increase confidence in the underlying algorithms you have written if there is input/output parity between valr and BEDTools, at least for the tools that overlap the two packages. For example I see some test cases that use input from the BEDTools test suite (e.g., test_cluster.r), but don't check the output. It should be straightforward to check the output against that provided by the BEDTools test suite. Correspondingly it would be good for BEDTools to use valr input/expected output in its test suite.

I have read this submission. I believe that I have an appropriate level of expertise to confirm that it is of an acceptable scientific standard.

# Peer review: Referee response for version 1

**DOI:** 10.5256/f1000research.12975.r23916

**Published:** 2017-07-10

**Authors:** Robert A. Amezquita

**Affiliations:** 1 Department of Immunobiology, School of Medicine, Yale University, New Haven, CT, USA

Riemondy and colleagues have made a valuable tool, `valr`, available to the general public, one which deserves much merit for bringing modern R idioms from the `tidyverse` into the world of genomic research. As a fellow bioinformatician who has struggled with the idiosyncrasies of the aforementioned tools for interval manipulation in R, `valr` addresses many of the usability issues associated with these legacy methods by fundamentally altering the user experience. Consequently, there are two main advantages to adopting `valr` for interval manipulation in R: ease of writing, and ease of reading, code. Thus, this referee wholeheartedly endorses `valr`, and hopes to see more work that brings many of the `tidyverse` philosophies over to working with genomics in R. 

 Nonetheless, there is some room for improvement of the associated manuscript to better help explain the philosophy and usage of `valr`, and its place amongst the many tools for the manipulation of genomic intervals.

 Firstly, in the introduction, it is mentioned that there exist `IRanges` methods that utilize the S4 convention, whereas `valr` utilizes a less formal schema where 3 columns, `chrom`, `start`, `end`, are present in the `data_frame` object. Indeed, it may be of use to expand upon such design choices that were made, and what advantages/disadvantages are made in using this less formal schema, and any other highly pertinent choices that affect user experience. In addition, one line mentions integration with other `tidyverse` tools, and should expand upon this with either one to a few specific examples or explain this point in more detail. Additionally, it should also be pointed out how `valr` builds upon these existing toolkits, and either expands upon/adopts their conventions. One way might be to create a table comparing functions between `valr`/bedtools/GenomicRanges might be helpful for a reader to see that the toolkit will be easily adoptable. Indeed, its mentioned that the syntax is similar to bedtools in the use cases, and might be good to mention in the introduction as well. Thus, an expanded introduction/additional section explaining the uniqueness of `valr` would help to better "sell" when one should use `valr` and why.

 In performing benchmarking, it would be useful to include one or two leading R tools, such as GenomicRanges, into the calculations, as this is likely how many R programmers currently perform interval manipulations natively in R, and I suspect would likely show an impressive performance improvement by relation.

 `valr` presents an exciting new development in the R+Genomics realm, and this referee is hopeful that this sort of development helps fuel further `tidyomics` tools for R bound together by a cohesive philosophy, great user experience, and pointed utility. 

I have read this submission. I believe that I have an appropriate level of expertise to confirm that it is of an acceptable scientific standard.
